# Pseudorabies Virus Regulates the Extracellular Translocation of Annexin A2 To Promote Its Proliferation

**DOI:** 10.1128/jvi.01545-22

**Published:** 2023-02-14

**Authors:** Maoyang Weng, Zhenhua Guo, Qingxia Lu, Qianyue Jin, Yao Jiang, Fangyu Wang, Junqing Guo, Guangxu Xing, Songlin Qiao, Gaiping Zhang

**Affiliations:** a College of Veterinary Medicine, Northwest A&F University, Yangling, Shaanxi, China; b Key Laboratory of Animal Immunology of the Ministry of Agriculture, Henan Provincial Key Laboratory of Animal Immunology, Henan Academy of Agricultural Sciences, Zhengzhou, Henan, China; c School of Advanced Agricultural Sciences, Peking University, Beijing, China; d Longhu Modern Immunity Laboratory, Zhengzhou, Henan, China; e Jiangsu Co-innovation Center for Prevention and Control of Important Animal Infectious Diseases and Zoonoses, Yangzhou, China; Lerner Research Institute, Cleveland Clinic

**Keywords:** pseudorabies virus, US3, annexin A2, Src, phosphorylation, antiviral, antiviral agents, protein phosphorylation

## Abstract

Pseudorabies virus (PRV) infection causes enormous economic losses to the pork industry and severe health consequences in many hosts. Annexin A2 (ANXA2) is a membrane-associated protein with various intracellular functions associated with many viral infections. However, the role of ANXA2 in alphaherpesvirus replication is still not explored. In the present study, we identified the interaction between ANXA2 and PRV US3. The deficiency of ANXA2 significantly restricted PRV proliferation. PRV infection or US3 overexpression led to ANXA2 extracellular translocation. Furthermore, we confirmed that PRV or US3 could lead to the phosphorylation of the Tyr23 ANXA2 and Tyr419 Src kinase, which was associated with the ANXA2 cell surface transposition. US3 can also bind to Src in an ANXA2-independent manner and enhance the interaction between Src and ANXA2. Additionally, inhibitors targeting ANXA2 (A2ti-1) or Src (PP2) could remarkably inhibit PRV propagation *in vitro* and protect mice from PRV infection *in vivo*. Collectively, our findings broaden our understanding of the molecular mechanisms of ANXA2 in alphaherpesvirus pathogenicity and suggest that ANXA2 is a potential therapeutic target for treating alphaherpesvirus-induced infectious diseases.

**IMPORTANCE** PRV belongs to the alphaherpesvirus and has recently re-emerged in China, causing severe economic losses. Recent studies also indicate that PRV may pose a potential public health challenge. ANXA2 is a multifunctional calcium- and lipid-binding protein implicated in immune function, multiple human diseases, and viral infection. Herein, we found that ANXA2 was essential to PRV efficient proliferation. PRV infection resulted in the extracellular translocation of ANXA2 through phosphorylation of ANXA2 and Src. ANXA2 and Src formed a complex with PRV US3. Importantly, inhibitors targeting ANXA2 or Src prevented PRV infection *in vitro* and *in vivo*. Therefore, our studies reveal a novel strategy by which alphaherpesvirus modifies ANXA2 to promote its replication and highlight ANXA2 as a target in developing novel promising antivirus agents in viral therapy.

## INTRODUCTION

Pseudorabies virus (PRV) is the causative agent of pseudorabies (PR), which is also known as Aujeszky’s disease (AD). PRV belongs to the family *Herpesviridae*, subfamily *Alphaherpesvirinae*, and genus Varicellovirus and is an enveloped virus with a large linear double-stranded DNA genome encoding more than 70 proteins (1). In addition to natural host pigs, PRV can infect multiple animals, including dogs, cats, rabbits, cattle, foxes, and wolves (2). Most other non-natural hosts die within 24 to 48 h of the disease onset, characterized by severe pruritus in the head and neck, accompanied by self-mutilation (3). Since 2011, PRV has been widely prevalent in pigs in China because of the emergence of PRV variants that can better evade the immune protections provided by the available commercial vaccines (4, 5). Recent studies suggest that these PRV variants could directly infect humans in particular conditions, raising the concern of PRV cross-species transmission (6–8). Thus, it is significantly important to study PRV pathogenic mechanisms and intensively explore potential antiviral targets.

The US3 serine/threonine kinase is conserved among the alphaherpesvirus family and represents an important virulence factor (9). Studies have shown that US3 can modify several biological processes of host cells to benefit PRV replication. For example, US3 triggers RhoA phosphorylation and cofilin dephosphorylation to reorganize the actin cytoskeleton (10, 11). US3 prevents host cells from apoptosis, disrupts the promyelocytic leukemia nuclear bodies (PML-NBs), downregulates the expression of histocompatibility complex (MHC) class I, and induces degradation of host protein Bclaf1 to interfere with host defense mechanisms (12–14). In herpes simplex virus 1 (HSV-1), US3 can interact with and hyperphosphorylate β-catenin and interferon (IFN) regulatory factor (IRF3), which blocks IFN-I production (15, 16). Thus, it would be helpful to identify novel substrates interacting with US3 to understand better the signaling networks affected by the US3 protein and the underlying mechanisms.

Annexin A2 (ANXA2) is multifunctional calcium- and lipid-binding protein that belongs to a large family with more than 160 members (17). ANXA2 exists as a monomer localized to the cytoplasm, vesicle bound, or in complex with S100A10 (also known as p11, here referred to as p11) to form the ANXA2/p11 heterotetramer (A2t), which was found on the inner and outer leaflet of the plasma membrane (18). ANXA2 and A2t have been implicated in various cellular functions such as endocytosis, exocytosis, membrane domain organization, and translational regulation (19). Additionally, many discoveries showed that ANXA2 is implicated in viral pathogenesis. ANXA2 can interact with Nsp9 of porcine reproductive and respiratory syndrome virus (PRRSV), NS1 of avian influenza virus (AIV), and E2 glycoprotein of classical swine fever virus (CSFV) to prompt viral replication in host cells (20–22). Moreover, ANXA2 can bind with capsid protein VP1 of Enterovirus71 (EV71) and enhance viral infectivity (23). ANXA2 and A2t were also discovered as central mediators of HPV entry and intracellular trafficking (24). In PRV, previous research showed that ANXA2 could be incorporated into mature PRV virions (25), and ANXA2 was also found to participate in efficient retrograde transport of PRV within neurons (26). However, the role of ANXA2 in PRV replication and the underlying mechanisms is still unclear.

Our study found that ANXA2 was a novel host protein interacting with PRV US3. Knockdown or knockout ANXA2 significantly restricted PRV proliferation. Furthermore, PRV infection or US3 overexpression could lead to ANXA2 extracellular translocation through phosphorylating ANXA2 and Src kinase. ANXA2, together with Src, formed a complex with US3. Particularly, specific inhibitors targeting ANXA2 or Src prevented PRV multiplication *in vitro* and protected mice from PRV infection *in vivo*. These findings reveal the mechanism of ANXA2 extracellular translocation mediated by PRV and highlight its potential therapeutic targets for alphaherpesvirus infectious diseases.

## RESULTS

### Identification of the interaction between ANXA2 and PRV US3.

**FIG 1:**
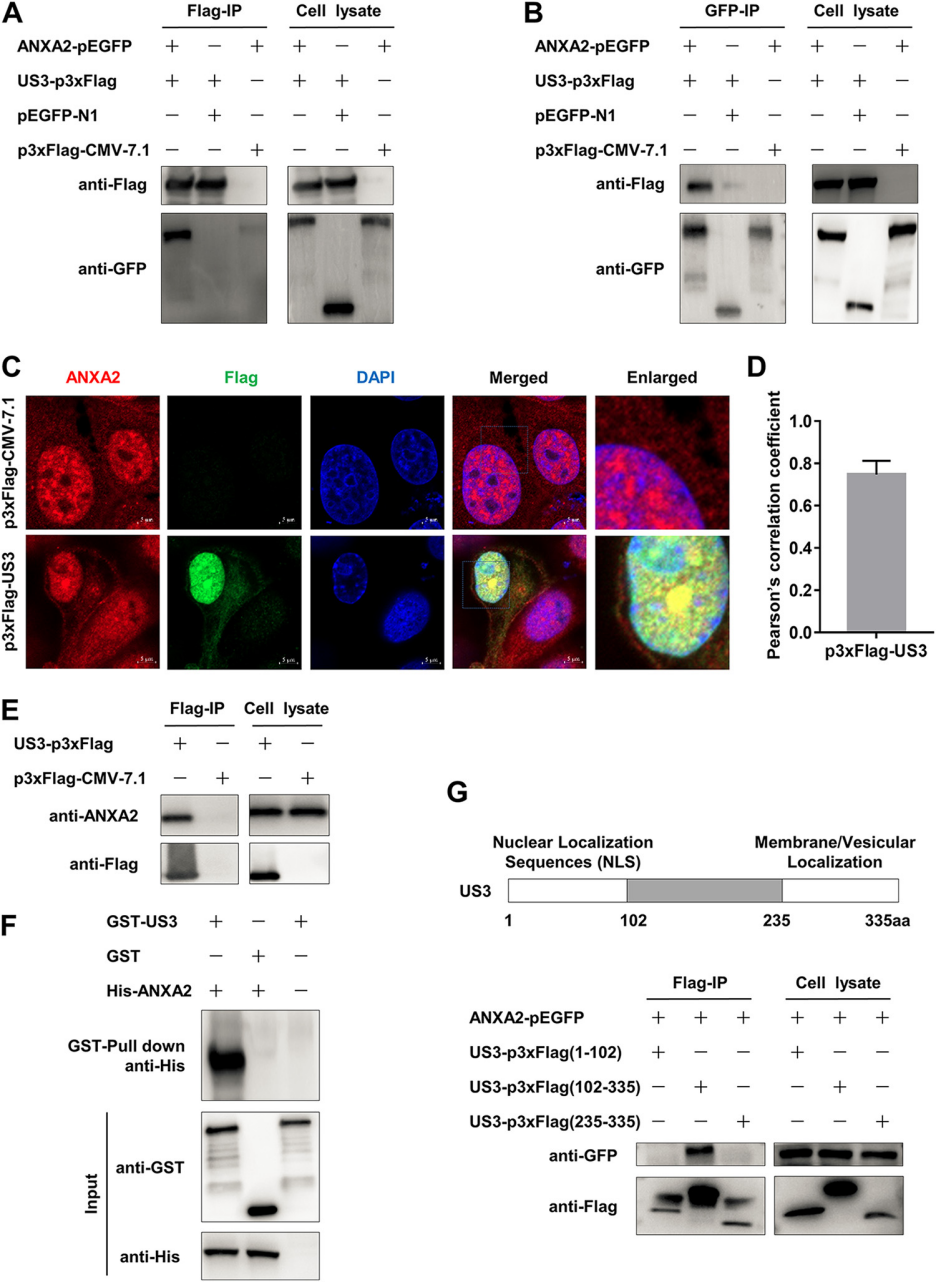
Identifying the interaction between annexin A2 (ANXA2) and pseudorabies virus (PRV) US3. (A, B) US3-p3×Flag interacted with exogenous ANXA2. The HEK-293T cells were coexpressed with recombinant US3-p3×Flag and ANXA2-pEGFP. Both Flag-IP and GFP-IP showed that exogenous US3 interacted with exogenous ANXA2. (C, D) US3-p3×Flag colocalized with endogenous ANXA2. PK-15 cells were transfected with US3-p3×Flag or p3×Flag-CMV-7.1 for 36 h and stained with primary anti-Flag MAb followed by Alexa Fluor 488 donkey anti-mouse antibody (green). Endogenous ANXA2 stained with primary anti-ANXA2 MAb followed by Alexa Fluor 647 donkey anti-rabbit antibody (red). The cell nuclei were stained with 4′,6-diamidino-2-phenylindole (DAPI) (blue). The colocalization was assessed by the determination of Pearson’s correlation coefficient. Scale bars, 5 μm. (E) The interaction between endogenous ANXA2 and overexpressed US3-p3×Flag. PK-15 cells were harvested 36 h posttransfected US3-p3×Flag and p3×Flag-CMV-7.1 plasmids. The proteins were immunoprecipitated in whole-cell lysate (WCLs) using anti-DYKDDDDK agarose beads and then separated by 12.5% SDS-PAGE, and their interaction was confirmed. (F) The recombinant ANXA2 is directly bound to US3. The recombinant proteins His-ANXA2 and GST-US3 were purified by BeaverBeads His tag and BeaverBeads GSH, respectively. Then GST-US3 coupled to GST beads, where GST served as control. Subsequently, the beads were incubated with His-ANXA2, and the eluted samples were subjected to Western blotting (WB) and detected by anti-His and anti-GST MAb. (G) Identification of US3 domains interacted with ANXA2. The HEK-293T cells were coexpressed with ANXA2-pEGFP and the three truncated US3-p3×Flag plasmids. Then the WCLs went through an IP assay with anti-DYKDDDDK agarose beads. aa, amino acids; CMV, cytomegalovirus; GFP, green fluorescent protein; GST, glutathione *S*-transferase; IP, immunoprecipitation; NLS, nuclear localization sequence.

As US3 plays a critical role in PRV infection (27), to explore the novel substrates interacting with US3, we used a liquid chromatography-tandem mass spectrometry (LC-MS/MS) by using US3-pEGFP-expressed in PK-15 cells. Meanwhile, the host factor ANXA2 was identified in the result of LC-MS/MS with a high score (Table S1). Next, recombinant US3-p3×Flag and ANXA2-pEGFP were coexpressed in HEK-293T cells, and coimmunoprecipitation (Co-IP) experiments showed that exogenous ANXA2 could interact with US3 (Fig. 1A and B). Confocal microscopy analysis also showed that endogenous ANXA2 colocalized with overexpressed US3-p3×Flag (Fig. 1C). The colocalization coefficient between ANXA2 and US3 was expressed as Pearson’s correlation coefficient, and the value was 0.79 (Fig. 1D), suggesting that there existed an interaction (value > 0.5) (28). Moreover, an immunoprecipitation (IP) assay was further performed, and the result indicated that US3 could bind to endogenous ANXA2 in PK-15 cells (Fig. 1E). Additionally, we conducted a glutathione *S*-transferase (GST) pulldown assay to confirm whether ANXA2 bind to US3 *in vitro*. GST-fused US3 (GST-US3) could combine with His-fused ANXA2 (His-ANXA2), which was not observed between GST and His-ANXA2 (Fig. 1F). As we know, US3 contains a nuclear localization sequence (NLS) (residues 1 to 102) and a membrane/vesicular localization sequence (residues 235 to 335) (29). To determine the regions of US3 responsible for binding to ANXA2, recombinant ANXA2-pEGFP and three truncated mutants of US3 were coexpressed, and an IP assay was performed. The result showed that residues 102 to 235 of US3 was sufficient to interact with ANXA2 (Fig. 1G). Collectively, these results demonstrate that ANXA2 can directly interact with PRV US3 protein.

### ANXA2 deficiency restricts PRV proliferation in 3D4/21 and PK-15 cells.

**FIG 2:**
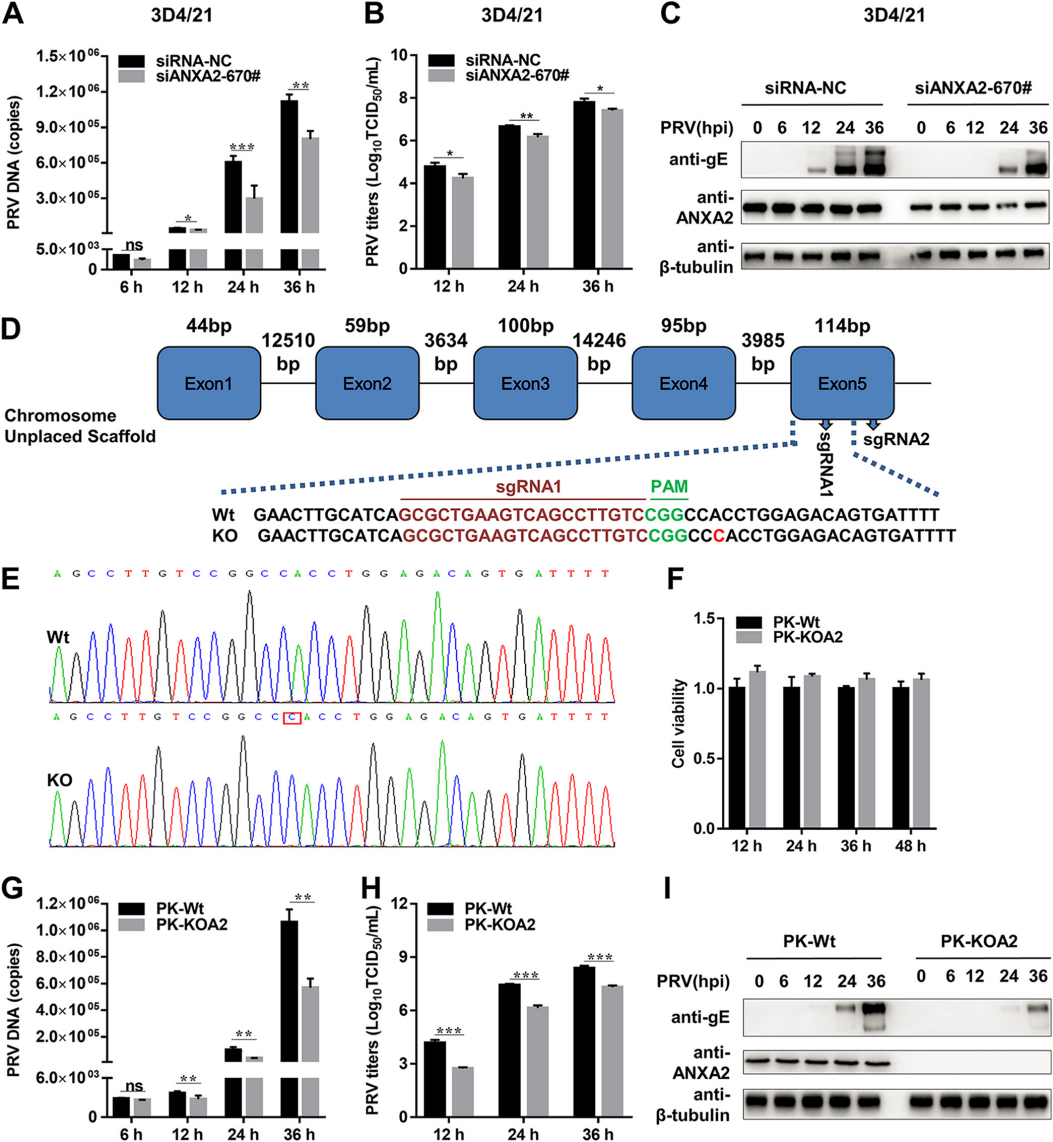
ANXA2 deficiency restricts PRV proliferation in 3D4/21 and PK-15 cells. (A to C) Knockdown of ANXA2 inhibited PRV replication. The 3D4/21 cells were seeded into 24-well plates, transfected with 50 nM siANXA2-670# or siRNA-NC for 36 h, and then inoculated with PRV (multiplicity of infection [MOI] = 0.01) for 6, 12, 24, and 36 h. The infected cells were harvested to detect PRV DNA abundance using quantitative real-time PCR (qPCR) for UL54, PRV progeny viral titers by 50% tissue culture infective dose (TCID_50_), and endogenous ANXA2 and PRV gE protein expression by WB. (D) Sequences in ANXA2 targeted by sgRNAs. CGG (green) is the protospacer adjacent motif (PAM). The highlighted base (red) indicates an indel. (E) Sanger sequencing showing the indel in ANXA2 (red square). (F) Viability assay of ANXA2 knockout (KO) cells. 2 × 10^4^ cells were seeded into 96-well plates, and viability was measured at 12, 24, 36, and 48 h. (G to I) Knockout of ANXA2 inhibited PRV infection. The PK-Wt and PK-KOA2 cells were seeded into 24-well plates and then inoculated with PRV (MOI = 0.01) for 6, 12, 24, and 36 h. The cells were harvested for the detection of PRV replication, as described above. NC, negative control; siRNA, small interference RNA; Wt, wild-type.

Next, we explored the biological significance of ANXA2 to PRV multiplication in host cells. Three specific small interference RNAs (siRNAs) targeting ANXA2 were utilized to detect its function. Noncytotoxic ANXA2 knockdown by three siRNAs decreased ANXA2 RNA abundance (~90% reduction) and protein level (data not shown) in 3D4/21 cells. Then, we found that noncytotoxic ANXA2 knockdown by siANXA2-670# in 3D4/21 cells significantly decreased PRV DNA copies (Fig. 2A), progeny viral titers (Fig. 2B), and gE protein level (Fig. 2C). These results demonstrate that knockdown of ANXA2 restricts PRV proliferation.

To further confirm the effect of ANXA2 on PRV proliferation, we constructed an ANXA2 knockout PK-15 cell line (PK-KOA2) using CRISPR-Cas9 targeting its exon 5 (Fig. 2D). Sanger sequencing confirmed that the ANXA2 sequence had been disrupted successfully (Fig. 2E). Western blotting (WB) and indirect immunofluorescence assay (IFA) showed that ANXA2 levels in the knockout cells were below the limits of detection, and the generation of the knockout cell line was successful (data not shown). The CCK-8 cell counting assay indicated that ANXA2 knockout did not affect cell viability (Fig. 2F). Subsequently, the proliferation efficiency of PRV was assessed in PK-Wt and PK-KOA2 cells. PRV proliferation levels in PK-KOA2 cells were significantly reduced compared to PK-Wt as determined by quantitative real-time PCR (qPCR) for UL54 (Fig. 2G), 50% tissue culture infective dose (TCID_50_) assay (Fig. 2H), and WB for gE protein expression (Fig. 2I). Taken together, these results suggest that ANXA2 is essential for PRV efficient proliferation.

### ANXA2 involves PRV release during the infection of host cells.

**FIG 3:**
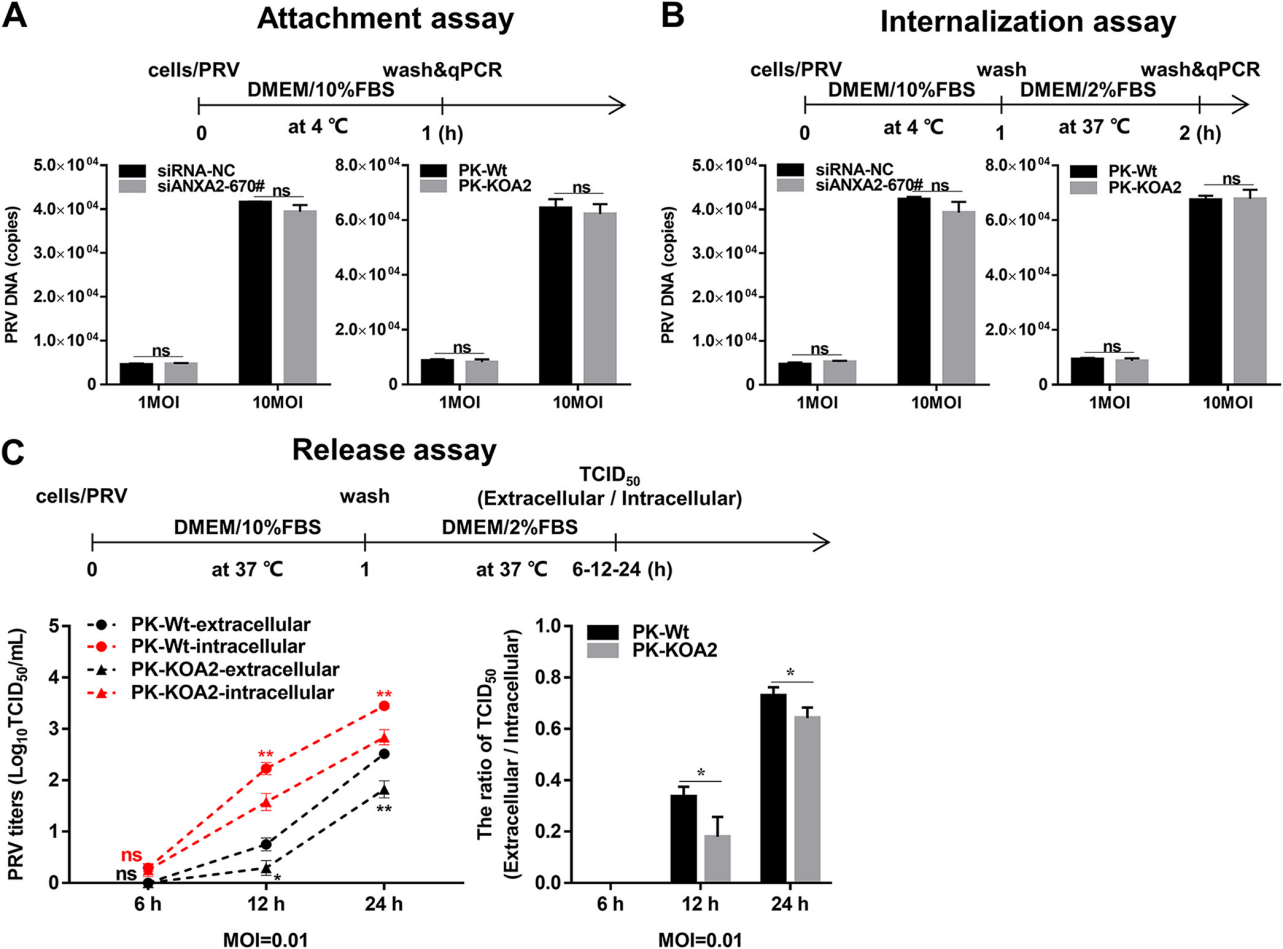
ANXA2 involves PRV release during the infection of host cells. (A, B) Attachment and internalization assay. 3D4/21 (siANXA2-670# and siRNA-NC) or PK-15 (PK-Wt and PK-KOA2) cells were incubated with PRV (MOI = 1 and 10) for 1 h at 4°C. After washing with cold phosphate-buffered saline (PBS) three times, the cells were harvested to extract the DNA for attachment assay, whereas the cells were further cultured in Dulbecco’s modified Eagle’s medium (DMEM) with 2% fetal bovine serum (FBS) for 1 h at 37°C for internalization assay. Both were assessed using qPCR for UL54. (C) Release assay. PK-Wt and PK-KOA2 cells were incubated with PRV (MOI = 0.01) for 1 h at 37°C, and then the cells were washed and inoculated with 2% FBS DMEM at 37°C. The cells and supernatants were harvested at 6, 12, and 24 h, respectively. Both the extracellular and intracellular virus titers at different time points were evaluated by TCID_50_. The ratio of extracellular and intracellular TCID_50_ suggested that ANXA2 participated in regulating the release process during PRV infection.

Based on findings that ANXA2 can interact with US3 and affect PRV proliferation, we further investigated the effects of ANXA2 on PRV attachment, internalization, and release. Notably, compared with the control cells, there were no significant differences in PRV attachment (Fig. 3A) and internalization (Fig. 3B) in the ANXA2 knockdown or knockout cells. However, the multiplication of PRV progeny virus, both extracellularly and intracellularly, was decreased significantly in PK-KOA2 cells than in PK-Wt cells (Fig. 3C). Importantly, the ratio of viral titer between extracellular and intracellular was significantly smaller in PK-KOA2 cells than in PK-Wt cells (Fig. 3C), which suggested that ANXA2 deficiency impaired the release of PRV progeny virus. These data show that ANXA2 also participates in the release process to some extent during PRV replication.

### PRV infection leads to extracellular translocation of ANXA2.

**FIG 4:**
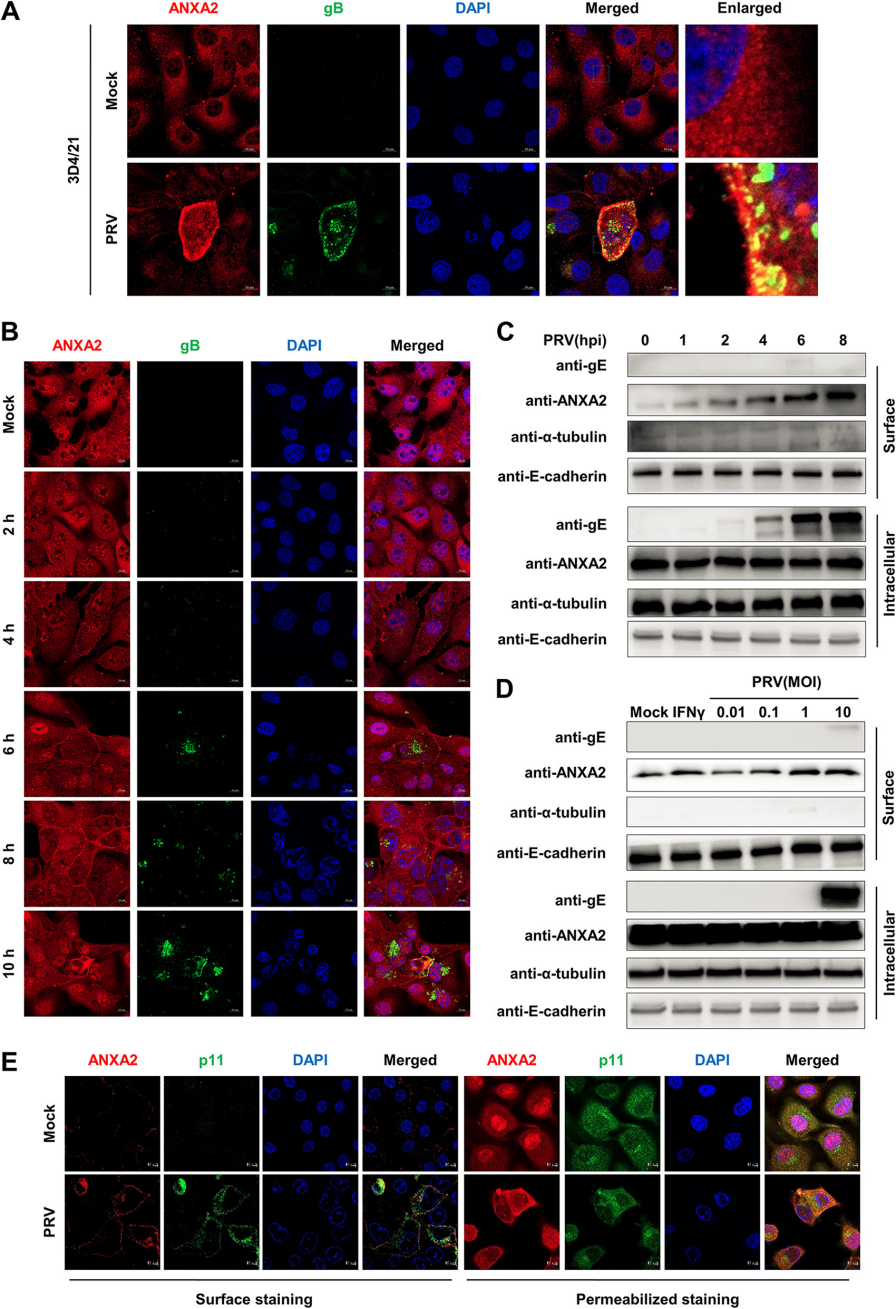
PRV infection leads to extracellular translocation of ANXA2. (A) PRV infection resulted in cell-surface translocation of ANXA2 in 3D4/21 cells. The cells were infected with PRV (MOI = 0.1) for 12 h, fixed with 4% PFA, permeabilized with 0.1% Triton X-100, and then incubated with primary ANXA2 monoclonal antibody (MAb) and gB MAb, followed by Alexa Fluor 647 donkey anti-rabbit antibody (red) and Alexa Fluor 488 donkey anti-mouse antibody (green). After nuclear staining with DAPI (blue), ANXA2, gB, and DAPI were observed by confocal microscopy. Scale bars, 10 μm. (B to D) PRV infection-induced surface translocation of ANXA2 in PK-15 cells. PRV (MOI = 10) cells were infected at the indicated time points (2, 4, 6, 8, and 10 hours). Then confocal microscopy analysis was performed as described above. Scale bars, 10 μm. Meanwhile, the cells were infected with PRV (MOI = 10) at the indicated time points (1, 2, 4, 6, and 8 hours) or with PRV (MOI = 0.01, 0.1, 1, and 10) at 6 h. Interferon-γ (IFN-γ) was used as a positive irritant, and the cell surface was eluted with 20 mM EGTA for 30 min at 4°C. The surface ANXA2 from EGTA eluates, and intracellular ANXA2 from cell lysates were analyzed by WB with α-tubulin and E-cadherin as controls. (E) PRV infection induced surface translocation of p11 in PK-15 cells. The cells were infected with PRV (MOI = 10) at 6 h; for surface staining, the nonfixed cells were stained with anti-p11 MAb and anti-ANXA2 Mab and then fixed with 2% PFA and stained with Alexa 647- or 488-conjugated secondary antibodies; for permeabilized staining, the cells were fixed with 4% PFA and permeabilized with 0.1% Triton X-100 and then stained for proteins. ANXA2, p11, and DAPI were observed by confocal microscopy. Scale bars, 10 μm.

We next explored the effect of PRV infection on ANXA2. Our data showed that the expression of ANXA2 was almost unchanged regardless of RNA abundance or protein level during PRV infection in PK-15 cells (data not shown). However, we observed obvious plasma membrane distribution of ANXA2 in PRV-infected 3D4/21 cells (Fig. 4A). Similar results were also confirmed in PK-15 cells infected with PRV by confocal microscopy (Fig. 4B). Previous studies showed that ANXA2 would translocate to the external leaflets of the plasma membrane upon cell exposure to certain stimuli (30). Thus, we further sought to know whether PRV infection can induce the extracellular translocation of ANXA2 following the studied method (24). We used EGTA (an extracellular calcium chelator) to release ANXA2, which is calcium-dependently bound to the cell surface (31). The level of cell surface ANXA2 in the EGTA eluate was increased by PRV infection at different time points (Fig. 4C) and doses (Fig. 4D). E-cadherin, as a control protein in the EGTA eluate, quickly migrates to cell membranes once synthesized and is easily eluted by EGTA due to its specific binding with Ca^2+^ (32). IFN-γ, which has been verified to induce surface translocation of ANXA2 (31), was used as a positive control. Additionally, we observed that the p11 protein, a partner of ANXA2 trafficking, was also increased in the cell surface after PRV infection, in both nonpermeabilized (Fig. 4E, left) and permeabilized PK-15 cells (Fig. 4E, right). Altogether, these results show that PRV infection can promote the extracellular translocation of ANXA2.

### PRV US3 induced extracellular translocation of ANXA2 in PK-15 cells.

**FIG 5:**
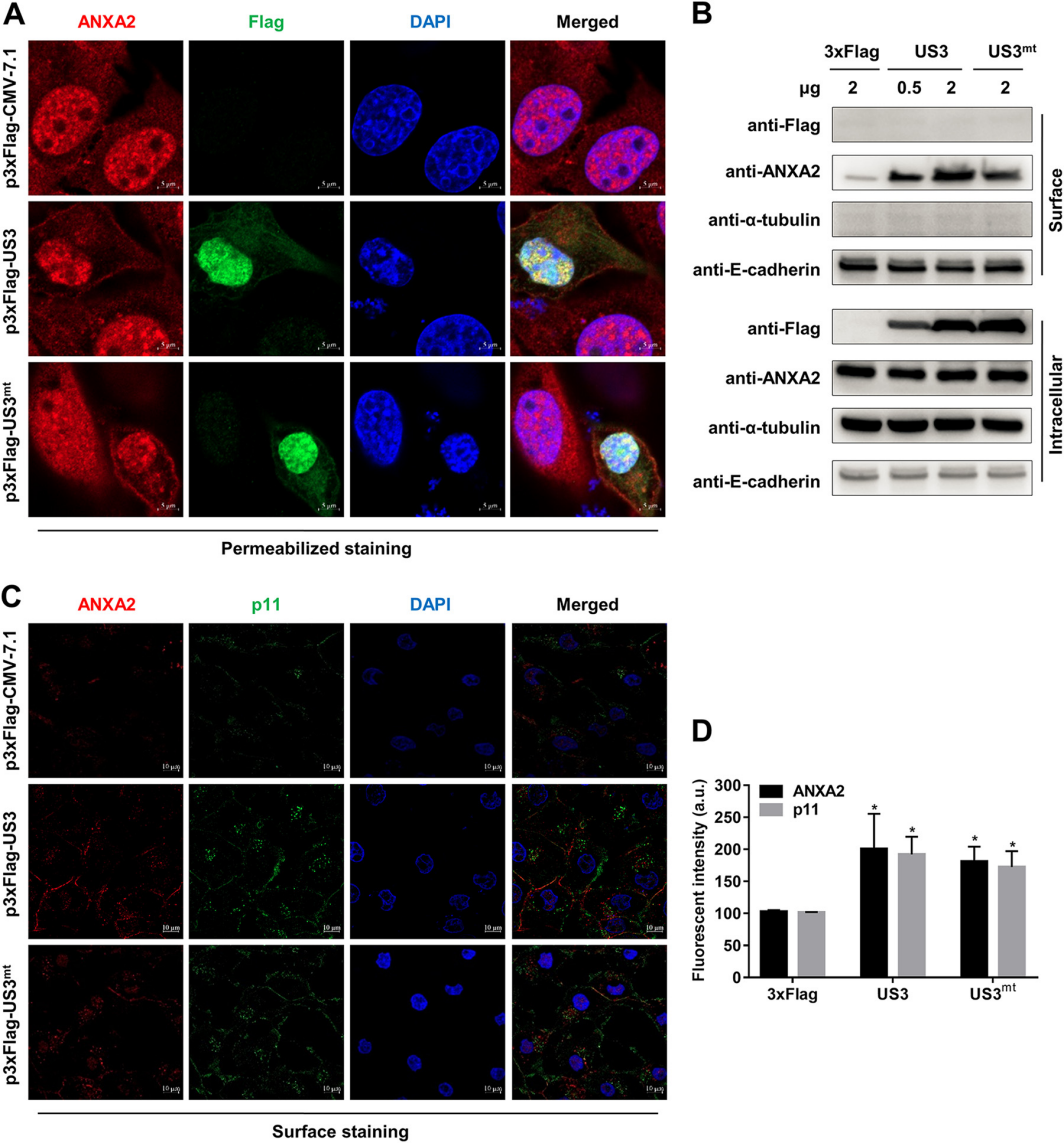
PRV US3 induced extracellular translocation of ANXA2 in PK-15 cells. (A, C) PRV US3 induced extracellular translocation of ANXA2 and p11 independent of its kinase activity. The cells were transfected with the same concentration of p3×Flag-CMV-7.1, US3, and US3^mt^-p3×Flag (2 μg) for 36 h, following permeabilized and surface staining. ANXA2, Flag, p11, and DAPI were observed by confocal microscopy. Scale bars, 5 and 10 μm. (B) Cells were transfected with p3×Flag-CMV-7.1 (2 μg), US3^mt^-p3×Flag (2 μg), and different concentrations (0.5 and 2 μg) of US3-p3×Flag for 36 h. The surface ANXA2 in the EGTA eluate was increased by US3-p3×Flag transfection at different doses. (D) The fluorescence intensity of ANXA2 or p11-expressing region in surface staining (C) was calculated using ImageJ software.

Since the US3 can interact with ANXA2 and US3 is a Ser/Thr kinase, we wondered whether US3 could also cause the increase of ANXA2 surface translocation and whether the kinase activity of US3 is involved. To generate the US3 kinase-inactive mutants (US3^mt^), we changed the valine at position 82 to glycine and the aspartic acid at position 169 to alanine, according to a previous study (33). Confocal microscopic observation confirmed that overexpression of US3 or US3^mt^ could increase the plasma membrane distribution of ANXA2 and p11 in PK-15 cells (Fig. 5A, C, and D). Accordingly, the protein quantity of surface ANXA2 in the EGTA eluate was also increased by US3- or US3^mt^-p3×Flag transfection in a dose-dependent manner (Fig. 5B), which was consistent with PRV infection. Collectively, our data suggest that both US3 and US3^mt^ can cause the extracellular translocation of ANXA2, which means that the kinase activity of US3 is not required for this process, and US3 may function as an adaptor.

### PRV infection and US3 protein result in the phosphorylation of ANXA2 and activation of Src kinase.

**FIG 6:**
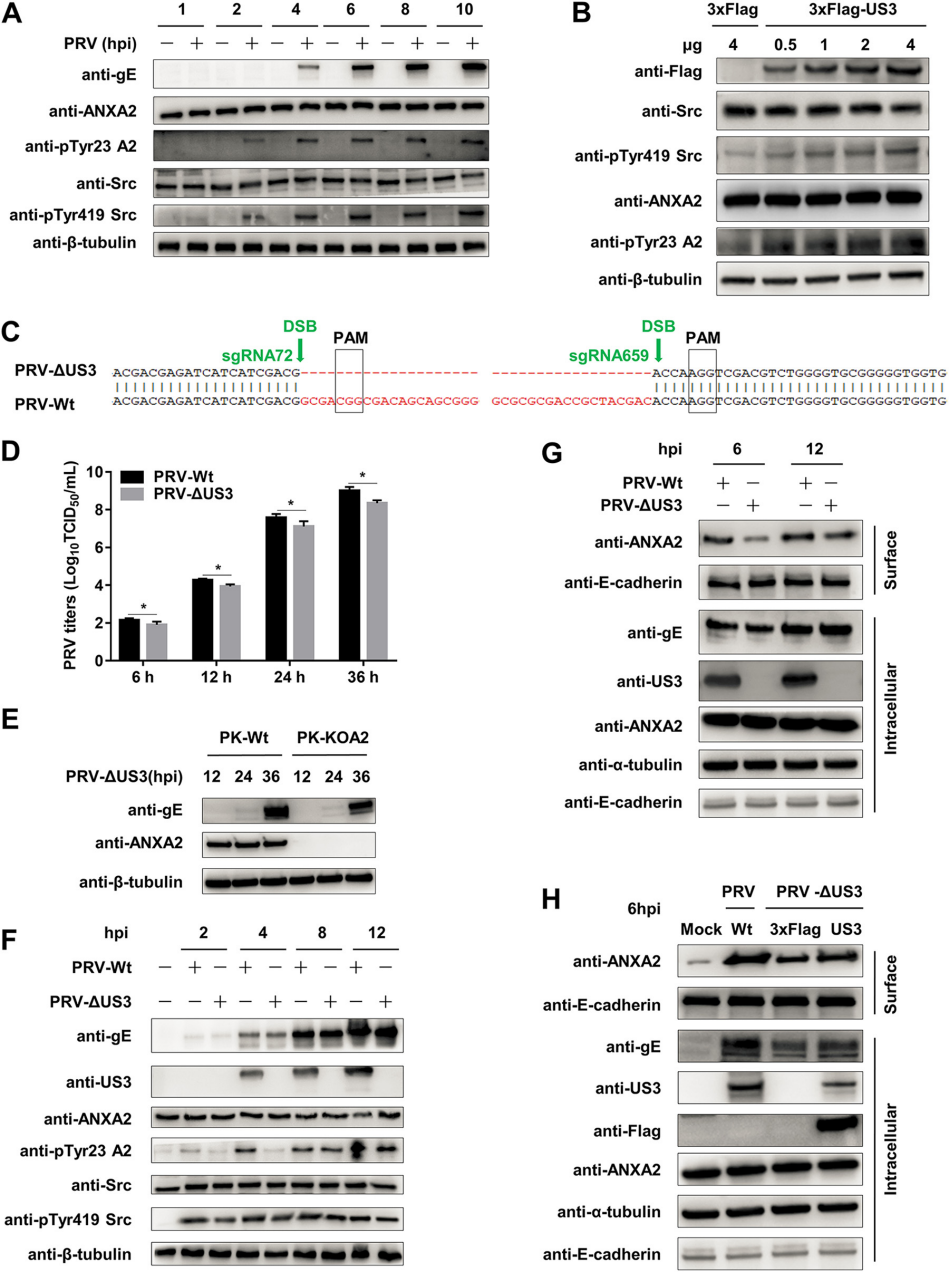
PRV infection and US3 protein result in the phosphorylation of ANXA2 and activation of Src kinase. (A) Tyrosine phosphorylation of ANXA2 and Src by PRV infection. PK-15 cells were collected postinfected with PRV (MOI = 10) or mock-infected at the indicated time points (1, 2, 4, 6, 8, and 10 h) and detected by WB. The signal of pTyr23 ANXA2 and pTyr419 Src was increased over time. (B) Tyrosine phosphorylation of ANXA2 and Src by PRV US3. The cells were transfected with p3×Flag-CMV-7.1 (4 μg) and different concentrations (0.5, 1, 2, and 4 μg) of US3-p3×Flag for 36 h. The levels of pTyr23 ANXA2 and pTyr419 Src also increased over time. (C) Sequences in PRV US3 targeted by sgRNA 72 and 659. CGG and AGG (gray rectangles) are the PAMs. (D) The growth curve of PRV-Wt and PRV-ΔUS3. PK-15 cells were collected postinfected with PRV-Wt or PRV-ΔUS3 (MOI = 0.01) at the indicated time points. The samples were harvested by repeated freezing and thawing three times and examined by TCID_50_ using Reed-Muench method. (E) Knockout of ANXA2 inhibited PRV-ΔUS3 infection. The PK-Wt and PK-KOA2 cells were collected postinfected with PRV-ΔUS3 (MOI = 0.01) for 12, 24, and 36 h. The infected cells were harvested to detect endogenous ANXA2 and PRV gE protein expression by WB. (F) The different phosphorylation efficiencies between PRV-Wt and PRV-ΔUS3. PK-15 cells were collected postinfected with PRV-Wt or PRV-ΔUS3 (MOI = 10) at the indicated time points (2, 4, 8, and 12 h), and the changes of pTyr23 ANXA2 and pTyr419 Src from cell lysates were analyzed by WB. (G) ANXA2 extracellular translocation induced by PRV-ΔUS3 was attenuated. PK-15 cells were infected with PRV-Wt or PRV-ΔUS3 (MOI = 10) at the indicated time points (6 and 12 h), and the differences between surface ANXA2 from EGTA eluates and intracellular ANXA2 from cell lysates were analyzed by WB with α-tubulin and E-cadherin as controls. (H) US3 supplementation contributed to the extracellular translocation of ANXA2 mediated by viral infection. PK-15 cells were transfected with p3×Flag-CMV-7.1 and US3-p3×Flag (4 μg) for 36 h, and the cells were collected postinfected with PRV-Wt or PRV-ΔUS3 (MOI = 10) for 6 h. The differences between surface ANXA2 from EGTA eluates and intracellular proteins from cell lysates were analyzed by WB. DSB, double-stranded break; hpi, hours postinfection.

Previous studies have shown that phosphorylation at Tyr23 of ANXA2 is important for its extracellular translocation, and ANXA2 is a substrate of Src kinase (34, 35). Therefore, we next wanted to know whether PRV infection can modify the phosphorylation of ANXA2 and Src kinase activity. Indeed, we found that the levels of pTyr23 ANXA2 and pTyr419 Src were significantly increased following PRV exposure at different periods (Fig. 6A), the same as PK-15 cells transfected with increased doses of US3-p3×Flag (Fig. 6B). To further confirm the regulatory function of US3, we constructed an US3-deleted PRV strain (PRV-ΔUS3) using CRISPR-Cas9 (Fig. 6C). PRV-ΔUS3 was verified by PCR assay with specific primers following DNA sequencing (data not shown). A mouse polyclonal antibody against US3 was also prepared to detect US3 protein. We first determined the effect of an absence of US3 on PRV replication and observed only minor titer reduction compared with PRV wild-type strain (Fig. 6D) in PK-15 cells, which is similar to a previous study (36). Then, the proliferation efficiency of PRV-ΔUS3 was assessed in PK-Wt and PK-KOA2 cells (Fig. 6E). Subsequently, we investigated the phosphorylation of ANXA2 and Src in PK-15 cells induced by wild-type or US3-deficient PRV. Our data showed that the pTyr23 ANXA2 induced by PRV-ΔUS3 was remarkably delayed in comparison to that induced by wild-type PRV, whereas the change of pTyr419 Src was slight or indistinguishable, implying that there were other viral proteins involved in the regulatory function of Src kinase (Fig. 6F). In line with it, PRV-ΔUS3 infection substantially decreased the ANXA2 extracellular translocation comparing with PRV wild-type strain (Fig. 6G). In addition, we also performed a US3 rescue experiment using wild- and ΔUS3-type PRV-infected cells (Fig. 6H), which suggested that US3 supplementation contributed to the extracellular translocation of ANXA2 to a certain extent. These results demonstrate that PRV infection and US3 induce ANXA2 phosphorylation at Tyr23 and activate Src kinase activity.

### PRV US3 interacts with Src kinase in an ANXA2-independent manner.

**FIG 7:**
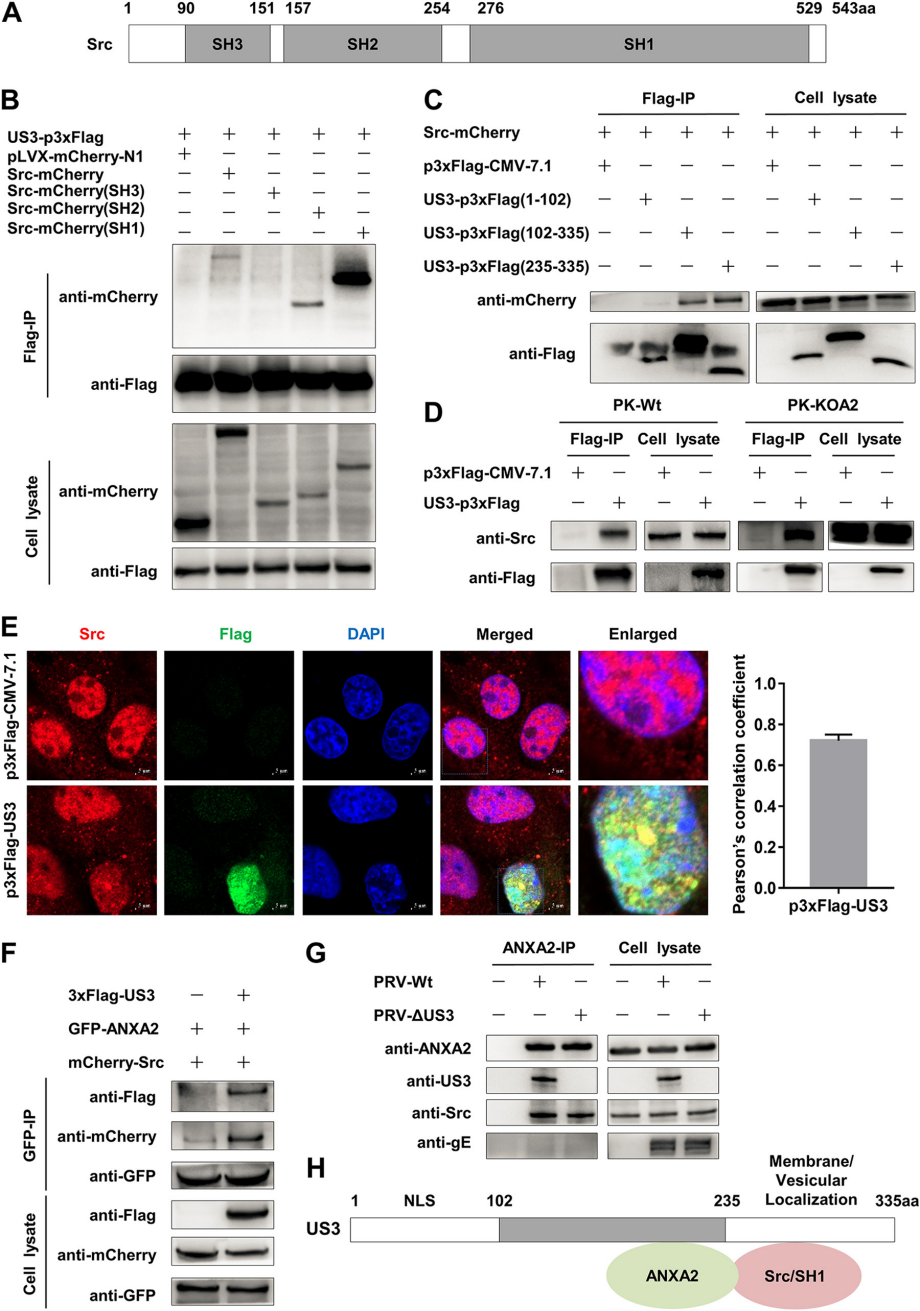
PRV US3 interacts with Src kinase in an ANXA2-independent manner. (A) Schematic diagram of the structure of porcine Src kinase. (B) Interactions between US3 and Src’s full-length or truncated fragments. The HEK-293T cells were coexpressed with US3-p3×Flag and the full-length or truncated fragments of Src-mCherry plasmids. All samples were immunoprecipitated from WCLs by anti-DYKDDDDK agarose beads. The immunoprecipitated proteins were immunoblotted with anti-Flag MAb and anti-mCherry polyclonal antibody. (C) Interactions between Src and the truncated fragments of US3. The HEK-293T cells were coexpressed with Src-mCherry and the corresponding empty vector or truncations of US3-p3×Flag. The samples were immunoprecipitated from WCLs by anti-DYKDDDDK agarose beads and then detected by WB. (D) The interaction between Src and US3 was ANXA2-independent. PK-Wt or PK-KOA2 cells were harvested 36 h posttransfected with US3-p3×Flag and p3×Flag-CMV-7.1 and then went through an IP assay. (E) Endogenous Src colocalized with US3. PK-15 cells were transfected with US3-p3×Flag and p3×Flag-CMV-7.1 plasmids for 36 h and stained with primary anti-Flag MAb followed by Alexa Fluor 488 donkey anti-mouse antibody (green). Src stained with primary anti-Src MAb followed by Alexa Fluor 647 donkey anti-rabbit antibody (red). The cell nuclei were stained with DAPI (blue). Scale bars, 5 μm. The colocalization was assessed by the determination of Pearson’s correlation coefficient. (F) US3 enhanced the binding of ANXA2 to Src. The HEK-293T cells expressing ANXA2-pEGFP and Src-mCherry with or without US3-p3×Flag were analyzed by immunoprecipitation. WB analyzed anti-GFP immunoprecipitates with anti-mCherry and anti-Flag antibodies. The cell lysates were carried out with WB assay with anti-Flag, anti-mCherry, or anti-GFP antibodies. (G) The endogenous interactions between ANXA2, Src, and PRV US3 in the infected PK-15 cells. The cells were infected with PRV or PRV-ΔUS3 at 0.01 MOI for 24 h, and the samples were immunoprecipitated from WCLs by anti-IgG and anti-ANXA2 and then detected by WB. (H) Schematic diagram representing the main binding domains between PRV US3 and ANXA2 or Src.

Knowing that ANXA2 is a substrate of Src and ANXA2 can interact with US3, we speculated that US3 could also bind to Src to form a complex. Porcine Src kinase has three domains, including SH1 (276 to 529 aa), SH2 (157 to 254 aa), and SH3 (90 to 151 aa) (Fig. 7A). To clarify the interaction between US3 and Src, we coexpressed US3-p3×Flag and Src-mCherry or truncated derivatives in HEK-293T cells. The Co-IP assay showed that exogenous Src and the SH1 and SH2 domains could bind to PRV US3. The SH1 domain had the strongest interaction signal, implying that the SH1 region of Src was primarily responsible for their interaction (Fig. 7B). Furthermore, recombinant Src-mCherry and truncated US3-p3×Flag mutants were also coexpressed in HEK-293T cells and performed IP assay. Our experiments showed that the membrane/vesicular localization sequence (residues 235 to 335) of US3 was enough to interact with Src (Fig. 7C). Additionally, to elucidate whether the interaction between Src and US3 was associated with ANXA2, the Flag-IP assay was carried out in US3-p3×Flag-expressed PK-Wt or PK-KOA2 cells. The results indicated that US3 could bind to endogenous Src in both PK-Wt and PK-KOA2 cells, indicating that its interaction was ANXA2 independent (Fig. 7D). Confocal microscopy was also implemented and showed that exogenous Src (data not shown) and endogenous Src (Fig. 7E) colocalized with US3-p3×Flag in PK-15 cells but not the empty vector control. Pearson’s correlation coefficient was 0.75 (data not shown) or 0.72 (Fig. 7E), suggesting an interaction existed. In addition, we also observed that the expression of US3 enhanced the binding of ANXA2 to Src (Fig. 7F), suggesting that US3 functionally contributes to the association between Src kinase and ANXA2. More importantly, Co-IP assays using IgG and ANXA2 rabbit monoclonal antibody (MAb) suggested that US3 and Src could be detected in wild-type PRV-infected cells, whereas only Src was detected in the ΔUS3-type infection condition (Fig. 7G). Altogether, these data strongly support that US3 might be a bridge protein to recruit Src and form a complex with ANXA2 in host cells (Fig. 7H).

### A2ti-1 or PP2 prevents PRV proliferation *in vitro*.

**FIG 8:**
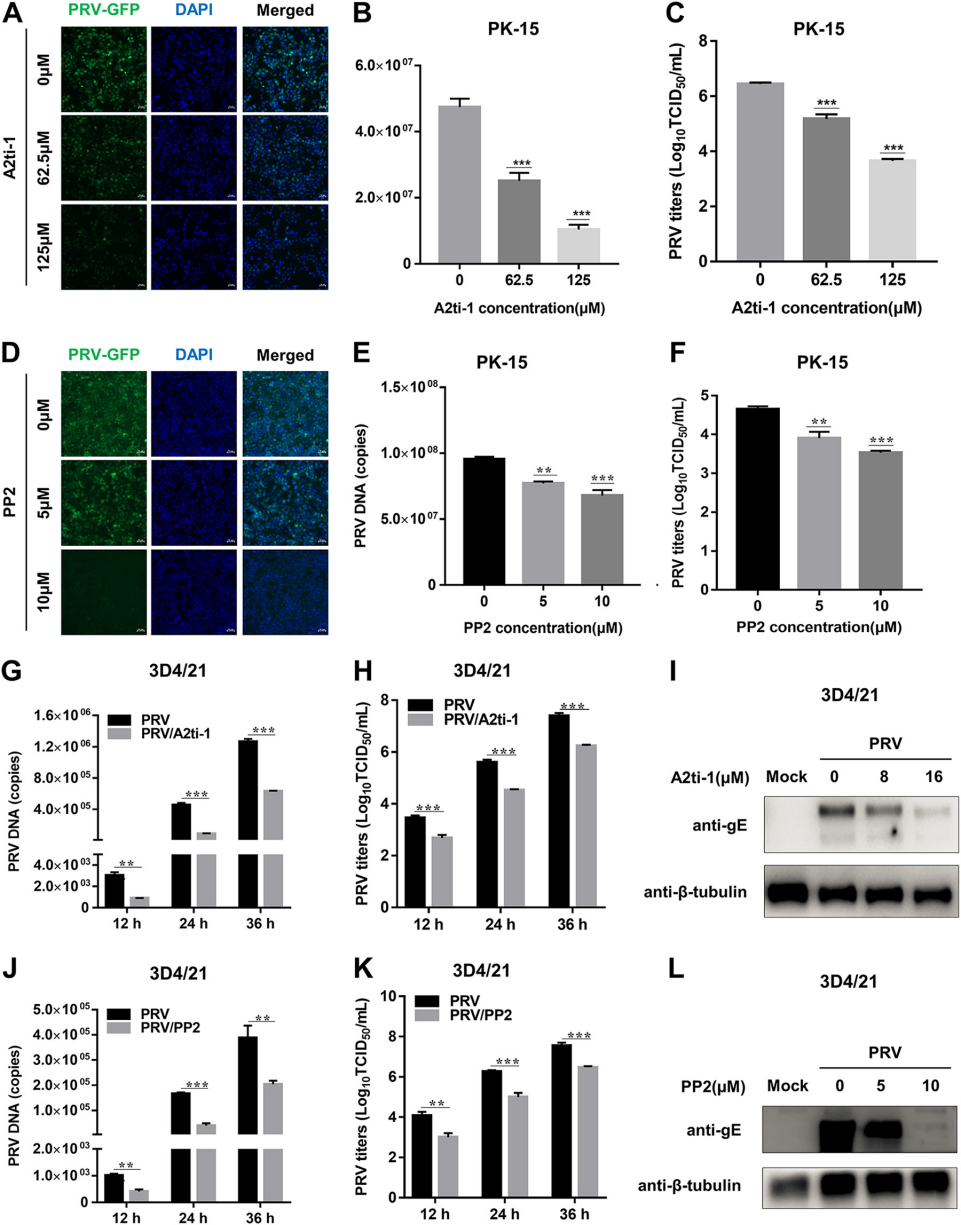
A2ti-1 or PP2 prevents PRV proliferation *in vitro*. (A, D) A2ti-1 or PP2 antagonized PRV-GFP infection by IFA. PK-15 cells grown in 96-well plates were incubated with PRV-GFP (MOI = 0.01) along with A2ti-1 (62.5 or 125 μM) or PP2 (5 or 10 μM) up to 24 hpi and then detected by immunofluorescence assay (IFA). Scale bars, 50 μm. (B, C, E, F) A2ti-1 or PP2 protected PK-15 cells from PRV infection in a dose-dependent manner. Cells grown in 24-well plates were incubated with PRV (MOI = 0.01) along with A2ti-1 or PP2 up to 24 hpi. The infected cells were harvested for detection of PRV DNA abundance using qPCR for UL54 and PRV progeny viral titers by TCID_50_. (G, H, J, K) A2ti-1 or PP2 protected 3D4/21 cells from PRV infection in a time-dependent manner. Cells grown in 24-well plates were incubated with PRV (MOI = 0.01) along with A2ti-1 or PP2 at the indicated time points. The infected cells were harvested for detection of PRV DNA abundance using qPCR for UL54 and PRV progeny viral titers by TCID_50_. (I, L) A2ti-1 or PP2 prevented PRV replication in a dose-dependent manner in 3D4/21 cells. Cells grown in 24-well plates were incubated with PRV (MOI = 0.01) along with A2ti-1 or PP2 up to 24 hpi. The cells were harvested to detect different kinds of endogenous protein expressions by WB.

Next, we used two inhibitors, A2ti-1 (target annexin A2/p11, A2t) and PP2 (target Src family kinases), to substantiate the antiviral effects of A2ti-1 or PP2 on PRV. To do this, we first assessed the cytotoxicity of A2ti-1 and PP2 using the CCK-8 cell counting assay in PK-15 cells (data not shown). Then, noncytotoxic inhibitors were utilized in PK-15 cells to examine their effects on PRV-GFP infection. Fluorescence microscopy analysis showed that A2ti-1 (62.5 or 125 μM) or PP2 (5 or 10 μM) could prominently restrict PRV-GFP proliferation (Fig. 8A and D). PRV viral DNA abundance and progeny virus production was also decreased in PK-15 cells treated with A2ti-1 (Fig. 8B and C) or PP2 (Fig. 8E and F) in a dose-dependent manner. Subsequently, we further evaluated whether the inhibitors affected the PRV replication in 3D4/21 cells. The cytotoxicity of inhibitors to 3D4/21 cells was first measured (data not shown). In line with the above studies, we found that PRV DNA abundance (Fig. 8G and J), progeny viral titers (Fig. 8H and K), and gE protein levels (Fig. 8I and L) were significantly decreased in A2ti-1- or PP2-treated cells compared with the nontreated cells, respectively. Collectively, our experiments demonstrate that inhibitors targeting ANXA2 or Src kinase can efficiently prevent PRV proliferation *in vitro*.

### A2ti-1 or PP2 protects mice from PRV infection *in vivo*.

**FIG 9:**
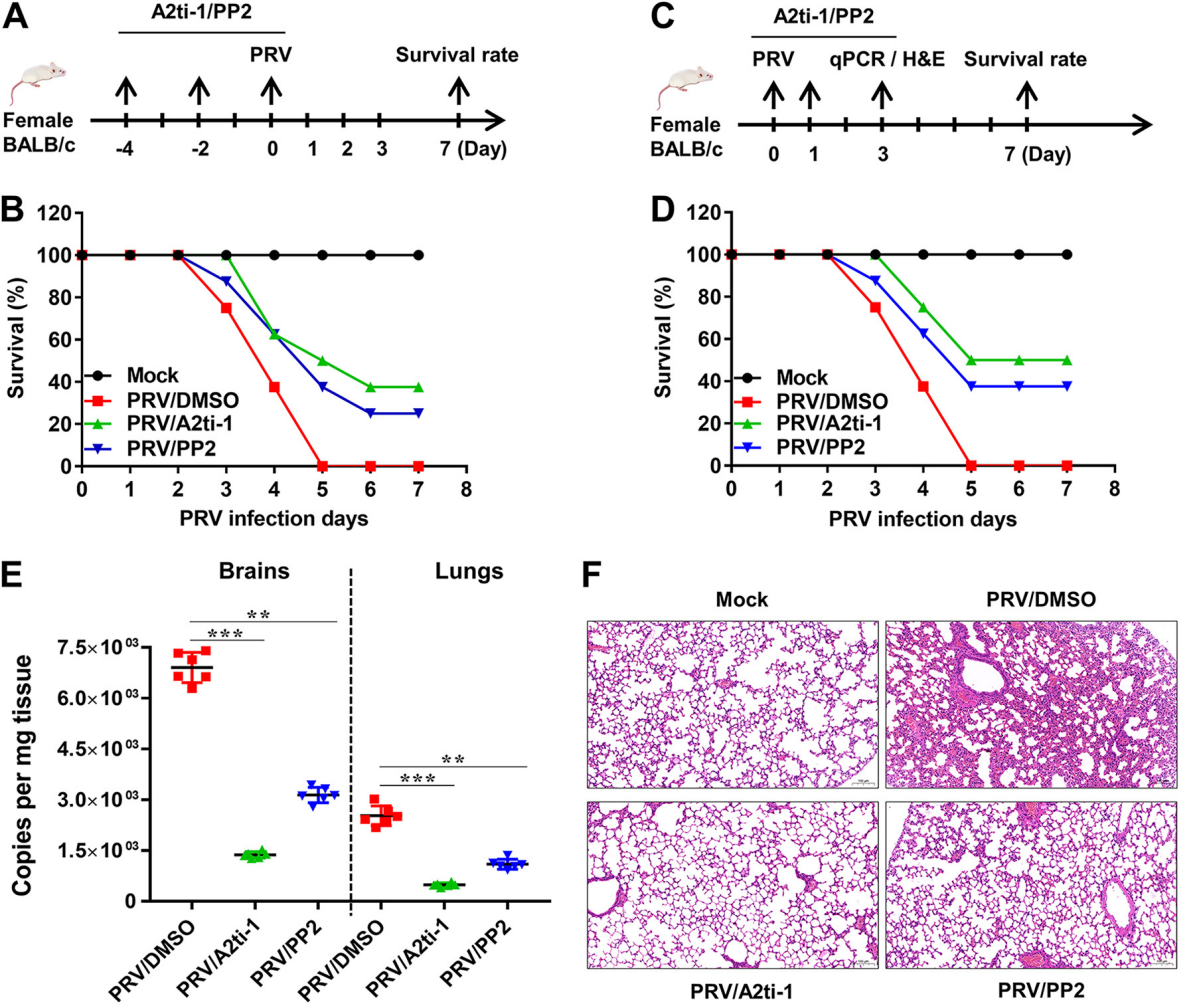
A2ti-1 or PP2 protects mice from PRV infection *in vivo*. (A, B) Preventive strategy for PRV challenge. Female BALB/c mice (*n* = 8) were intraperitoneally injected with dimethyl sulfoxide (DMSO) or A2ti-1 or PP2 (8 mg/kg) on days −4, −2, and 0. Meantime, mice were intranasally infected with PRV (6 × 10^3^ TCID_50_/mouse) on day 0. The survival rate was monitored daily for 7 days. Arrows represent the number and time of administration. (C, D) Therapeutic strategy for PRV challenge. On day 0, mice (*n* = 8) were intranasally infected with PRV (6 × 10^3^ TCID_50_/mouse). Mice were intraperitoneally injected with DMSO or A2ti-1 or PP2 (8 mg/kg) on days 0, 1, and 3. The survival rate was monitored daily for 7 days. Arrows represent the number and time of treatment. (E, F) Viral loads and pathological changes in therapeutic groups. On day 3, PRV genome copy numbers in brains and lungs were assessed by qPCR for UL54 (*n* = 3). Sections of the mouse and another half lungs were stained with hematoxylin-eosin staining (Servicebio, Wuhan, China). Scale bar, 100 μm. H&E, hematoxylin and eosin.

Based on the above results, we examined whether these inhibitors could be used as a potent antiviral against PRV infection *in vivo*. First, we determined the protective effect of A2ti-1 or PP2 on PRV infection *in vivo* (Fig. 9A). Our experiments showed that the mortality of mice injected with A2ti-1 or PP2 (8 mg/kg) was lower than that of mice injected with dimethyl sulfoxide (DMSO) (Fig. 9B). In addition, we also tested the therapeutic effect of A2ti-1 or PP2 on PRV infection (Fig. 9C). We found that the survival rate of mice injected with A2ti-1 or PP2 (8 mg/kg) was higher than that of mice injected with DMSO (Fig. 9D). Moreover, results from viral loads by qPCR for UL54 in different therapeutic groups of brains and lungs indicated that PRV genome copy numbers were decreased due to A2ti-1 or PP2 treatment, suggesting that A2ti-1 or PP2 inhibited PRV proliferation *in vivo* (Fig. 9E). Lung injury caused by PRV infection was significantly attenuated upon A2ti-1 or PP2 treatment in different therapeutic groups, and less infiltration of inflammatory cells was detected in the lungs in A2ti-1- or PP2-treated mice than in DMSO-treated mice (Fig. 9F). Altogether, these results indicate that A2ti-1 or PP2 might be a potential antiviral drug against alphaherpesvirus.

## DISCUSSION

**FIG 10:**
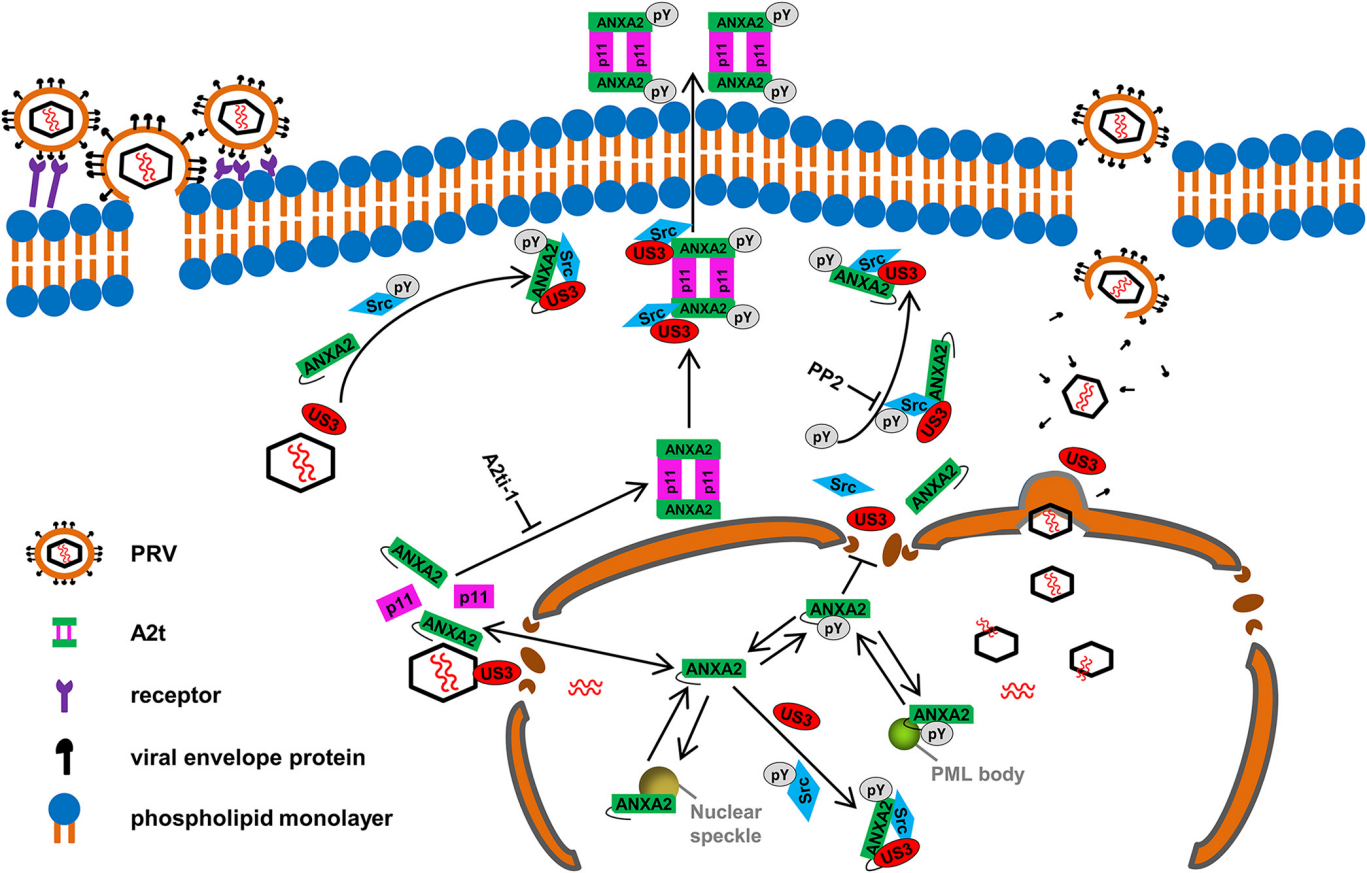
A schematic model showing the regulatory of ANXA2 extracellular translocation modified by PRV infection. ANXA2 is found in the nucleus and cytoplasm. ANXA2 usually exists as a monomer or complex with p11 to form the ANXA2/p11 heterotetramer (A2t) in the cytoplasm. p11 is believed to mobilize ANXA2 to the inner leaflets of the plasma membrane. Evidence shows that ANXA2 translocates to the cell surface upon exposure to certain stimuli. During PRV infection, once tegument protein US3 is exposed in the cytoplasm, it binds to ANXA2 and recruits Src kinase to form a complex, leading to the activation of Src kinase and Tyr23 phosphorylation of ANXA2. Following phosphorylation, ANXA2 in complex with p11 translocates to the external leaflets of the plasma membrane. In addition, we also found the colocalization of US3 with ANXA2 and Src in the nucleus. ANXA2 in the nucleus exists in both the nonphosphorylated and the Tyr23 phosphorylated form, showing differential association with different nuclear domains.

PRV is a swine alphaherpesvirus closely related to the human herpes simplex virus 1 (HSV-1) (37). The emergence and prevalence of PRV variants have led to huge economic losses in China (38). Recent evidence suggests that PRV can infect humans in particular conditions (6–8), which poses a potential challenge to public health security. In this study, we identified two novel host proteins, ANXA2 and Src, which formed a complex with PRV US3. The ANXA2 was essential for PRV efficient propagation. Furthermore, we revealed the underlying mechanism of ANXA2 extracellular translocation induced by PRV infection, which was first studied in alphaherpesvirus. We also found that the inhibitors targeting ANXA2 or Src displayed potential antiviral effects on alphaherpesvirus (Fig. 10).

US3 is a potent alphaherpesviral kinase that fulfills various cellular processes, including virus nuclear egress, cytoskeletal alteration, inhibition of apoptosis, and disruption of various host defense mechanisms (33, 36, 37, 39, 40). Some of these effects have been investigated mechanistically, and several phosphorylation targets of US3 have been identified. HSV-1 US3 can directly phosphorylate Lamin A/C, IRF3, TSC2, Beclin1, Bad, PKA, and KIF3A (41). PRV US3 has been shown to directly phosphorylate PAK1, PAK2, and histone deacetylases (HDACs) (10, 39). Here, we identified two novel host cell proteins, ANXA2 and Src kinase, which could bind to US3, and the US3 functionally conduces to the binding of ANXA2 to Src kinase. On the other hand, the colocalization of US3 with ANXA2 or Src was also observed in the nucleus. This is predictable since all three of these proteins also play multiple roles in the nucleus (13, 35, 42). For example, pTyr23 ANXA2 was observed to partially colocalize with PML-NBs, which the PRV US3 protein could disrupt (13, 35). Thus, the specific biological function of such interaction in the nucleus needs further elucidation. Furthermore, Tyr23 ANXA2 and Tyr419 Src phosphorylation was also observed in host cells with PRV infection or US3 overexpression, which was closely associated with the regulation of ANXA2 cell surface transportation. However, considering the US3 is a Ser/Thr kinase and our data also showed that the kinase activity of US3 was not essential for the extracellular translocation of ANXA2, we figured that US3 might activate the Src kinase activity through the structural changes caused by the protein interaction (42). Taken together, our results provide new insights into the signaling network of the US3 protein kinase, which broadens our knowledge of the functions of the alphaherpesvirus US3 protein.

ANXA2, an important member of the annexin family of proteins, involves many biological processes, such as endocytosis, exocytosis, autophagy, and cell-cell communications (43). Studies have shown that ANXA2 has been implicated in immune function, multiple human diseases, and viral infection (44, 45). ANXA2 can negatively regulate TLR4-triggered inflammatory responses through the TRAM-TRIF pathway (46). ANXA2 also attracted great attention from investigators to be an emerging biomarker and potential therapeutic target for aggressive cancers (43, 47). Additionally, ANXA2 was also found to play an implicated role in multiple life cycle steps of viral infection. ANXA2 is utilized by HPV, enterovirus 71 (EV71), respiratory syncytial virus (RSV), and cytomegalovirus (CMV) during cell attachment and penetration (22–24, 48, 49); by hepatitis C virus (HCV) and influenza A virus (IAV) during replication (20, 50); and by measles virus (MV) during assembly and maturation (51). In this experiment, knockdown or knockout ANXA2 expression could significantly inhibit PRV proliferation. We also observed that ANXA2 was partially involved in the release process of PRV since the ratio of viral titer between extracellular and intracellular was prominently lower in PK-KOA2 cells than in PK-Wt cells. However, the viral attachment and internalization were not affected by ANXA2, which is the first study of the role of ANXA2 in alphaherpesvirus replication. Furthermore, the important role of ANXA2 in human health and disease has prompted the development of pharmacological inhibitors of ANXA2 and A2t. For example, small molecule inhibitor A2ti-1 targets annexin A2/p11 heterotetramer (A2t) and has been shown to block HPV infection in HeLa cells (24). ANXA2 antibodies impair productive HIV-1 infection of macrophages *in vitro* (52). Our results also showed that inhibitor A2ti-1 could effectively inhibit PRV propagation *in vitro* and protect mice from PRV infection *in vivo*. Beyond that, PP2, a reversible and ATP-competitive Src family kinase (SFK) inhibitor, also showed a similarly antiviral effect. These studies suggested that ANXA2 was a potential target for developing a novel antivirus agent for alphaherpesvirus.

In summary, our results reveal that the ANXA2 is essential to PRV efficient replication and illustrate the molecular mechanism of PRV-mediated ANXA2 extracellular translocation. Our findings extend the understanding of the viral host interaction and contribute to the rational design of antivirus agents for alphaherpesvirus.

## MATERIALS AND METHODS

### Mice.

Female 5-week-old BALB/c mice were purchased from the Henan Scobes Biotechnology Co., Ltd. (Zhengzhou, China) and maintained in a specific pathogen-free animal facility according to the guide for the care and use of laboratory animals and the related ethical regulations at Henan Provincial Key Laboratory of Animal Immunology.

### Cells and viruses.

3D4/21 cells (American Type Culture Collection, ATCC, CRL-2843) were maintained in RPMI 1640 (Solarbio, 31800) supplemented with 10% heat-inactivated fetal bovine serum (FBS) (Gibco, 10270106) and antibiotics (penicillin 100 U/mL and streptomycin 100 mg/mL) (Solarbio, P1400). PK-15 (ATCC, CCL-33) and HEK293T (ATCC, CRL-11268) were routinely maintained in Dulbecco’s modified Eagle’s medium (DMEM) (Solarbio, 12100) also supplemented with FBS and antibiotics. All cells were grown in monolayers at 37°C in 5% CO_2_. The PRV virulent strain HeNLH/2017 (GenBank no. MT775883) was kept by our laboratory. The recombinant strain PRV-GFP was kindly donated by Hanzhong Wang from the Wuhan Institute of Virology, Chinese Academy of Sciences.

### Antibodies.

The antibodies anti-annexin A2 (8235), anti-GFP (2956), anti-Flag (14793), anti-GST (2624), anti-His (9991), anti-α-tubulin (2144), anti-β-tubulin (86298), and anti-E-cadherin (3195) were all purchased from Cell Signaling Technology; anti-p-annexin A2 (85.Tyr24) (sc-135753) was purchased from Santa Cruz Biotechnology; anti-mCherry (GTX128508) was purchased from GeneTex; anti-Src (T56605), anti-p-Src (T55468), and anti-S100A10/p11 (MB0140) were purchased from ab-mart. Alexa Fluor 488 donkey anti-mouse antibodies (A21202) and 647 donkey anti-rabbit antibodies (A31573) were purchased from Invitrogen. Anti-PRV gB and gE were stocked in our laboratory.

### Chemical reagents.

4′,6-Diamidino-2-phenyl-indole (DAPI) (C0065) was purchased from Solarbio (Beijing, China). ChromoTek GFP-Trap (gta) and DYKDDDDK Fab-Trap (ffa) were purchased from Proteintech (Wuhan, China). BeaverBeads GSH (70601-K10) and His tag (70501-K10) were purchased from Beaverbio (Suzhou, China). TRIzol reagent (15596018), Lipofectamine 2000 (11668019), and Lipofectamine RNAiMAX (13778150) were purchased from Thermo Fisher Scientific (Waltham, MA, USA). A2ti-1 (HY-136465) and PP2 (HY-13805) were purchased from MedChemExpress (South Brunswick, NJ, USA).

### RNA interference.

All siRNA-negative controls (NC) and siRNAs were designed and synthesized by GenePharma (Shanghai, China). In knockdown experiments, PK-15 or 3D4/21 cells were transfected with the indicated siRNAs at a final concentration of 50 nM using Lipofectamine RNAiMAX transfection reagent according to the manufacturer’s instructions for 36 or 48 h. Transfected cells were applied for subsequent experiments after cell viability measurement by a cell counting kit-8 (CCK-8) according to the instructions. The indicated siRNAs sequences are listed in Table S2.

### qPCR assay.

Viral DNAs were extracted by FastPure Viral DNA/RNA minikit (Vazyme, RC311-C1) or FastPure cell/tissue DNA isolation minikit (Vazyme, DC102-01) following the instructions. Total RNAs were extracted with TRIzol reagent, and the reverse transcription cDNAs were prepared using the PrimeScript RT Master Mix (TaKaRa, RR036B) and stored at –40°C. Viral genomic DNA was quantified using UL54-specific primers, which generated a 66-bp fragment of the viral gene UL54 after amplification (53). The cDNAs from different samples were performed with a FastStart Universal SYBR green master kit on a Roche LightCycler 480 system (Roche, Switzerland). The relative mRNA level was evaluated by the 2^−ΔΔCT^ method with glyceraldehyde-3-phosphate dehydrogenase (GAPDH) mRNA as an endogenous control (54). The indicated primers for qPCR analysis are listed in Table S2.

### Plasmid construction and transfection.

PRV US3 and its truncations, a two-point mutation US3^mt^ (valine to glycine at position 82 and the aspartic acid to alanine at position 169) (33), were synthesized and cloned into p3×Flag-CMV-7.1; porcine ANXA2 was synthesized and cloned into pEGFP-N1; and porcine Src and its truncations were synthesized and cloned into pLVX-mCherry-N1. Two specific single guide RNAs (sgRNAs) targeting the fifth exon of the porcine ANXA2 gene and four specific sgRNAs targeting the PRV US3 gene were synthesized into pX459 v2.0. All recombinant vectors were constructed by GENEWIZ (Suzhou, China). For the transfection, the plasmids were transfected with Lipofectamine 2000 or LTX with Plus reagent or FuGENE HD transfection reagent according to the experimental purpose. The indicated sgRNAs are listed in Table S2.

### Generation of gene knockout cell lines using CRISPR-Cas9.

On day 0, wild-type PK-15 cells were seeded in a 6-well plate at 1 × 10^6^ cells/well. On day 1, the cells were transfected with 3 μg pig sgRNA1 or pig sgRNA2 or each 1.5 μg sgRNA1 and sgRNA2 using FuGENE HD transfection reagent. On day 3, the cells from the 6-well plate were digested and spread evenly into 10-cm culture dishes, each well for three dishes. On day 4, all 10-cm dishes were sequentially added by selection in a culture medium containing puromycin (4, 5, or 6 mg/mL) (Solarbio, P8230). From day 7, the operation on the fourth day was repeated after rinsing the 10-cm dishes with phosphate-buffered saline (PBS) every 3 days and continued for about half a month until the macroscopic monoclonal cell mass grew. Single clonal knockout cells were obtained by serial dilution and verified by Sanger sequencing and immunoblot analysis. The indicated primers for identifying wild-type and knockout type are listed in Table S2.

### Generation of PRV-ΔUS3 using CRISPR-Cas9.

On day 0, wild-type PK-15 cells were seeded in a 6-well plate. On day 1, the cells were transfected with each 1.5 μg PRV sgRNA24 and sgRNA654; sgRNA24 and sgRNA659; sgRNA72 and sgRNA654; or sgRNA72 and sgRNA659 using FuGENE HD transfection reagent. On day 3, the cells from the 6-well plate were infected by PRV (MOI = 0.01). On day 5, the plate was stored at –40°C and then frozen and thawed at least three times before centrifugation. Then, the supernatants were collected and passed through 0.22-μm filters (Sartorius, 16541). Part of the viral nucleic acid was extracted, and the full-length and sheared US3 fragments were detected. When the 6-well plate was covered with PK-15 cells, and the wells sequentially were inoculated with serially diluted viruses (10^−2^- to 10^−6^-fold), leaving a negative well. After multiple rounds of plaque purification (55), we purified PRV-ΔUS3, followed by Sanger sequencing and immunoblot analysis. The indicated primers for identifying wild- and ΔUS3-type viruses are listed in Table S2.

### Western blotting (WB).

The cells were harvested and lysed in RIPA lysis buffer after being rinsed with ice-cold PBS three times, supplemented with protease and phosphatase inhibitors, and incubated on ice for 30 min, followed by centrifugation at 13,000 rpm at 4°C for at least 15 min. Whole-cell lysates (WCLs) were normalized to equal amounts of α-tubulin or β-tubulin, loaded and separated by 10% to 15% gradient SDS-PAGE, and electrotransferred onto 0.22-μm polyvinylidene fluoride (PVDF) membranes (Millipore, ISEQ00010). The membranes were blocked in 5% skimmed milk at room temperature (RT) for 1.5 h and probed with the indicated primary antibodies at 4°C overnight. The membranes were washed five times with Tris-buffered saline containing 0.5% Tween 20 (TBST) and incubated with horseradish peroxidase (HRP)-labeled goat anti-mouse or rabbit IgG antibody at RT for 1.5 h. After five washes with TBST, the immunoreactive bands were visualized with an ECL reagent and imaged using a chemiluminescence imaging system (Vilber Fusion FX7, France).

### Coimmunoprecipitation.

For IP, PK-15 cells were transfected with mCherry or Src-mCherry-expressed plasmids and p3×Flag or US3-p3×Flag-expressed plasmids for 48 h. HEK-293T cells were cotransfected for Co-IP with relevant plasmids for 36 h. The cells were lysed in RIPA lysis buffer, and the total cellular proteins were incubated with anti-DYKDDDDK or anti-GFP agarose beads at 4°C overnight according to the instructions. The samples were washed with TBS more than five times, and potentially associated proteins were tested by WB using the indicated antibodies.

### *In vitro* affinity isolation assay.

The genes encoding porcine ANXA2 protein were synthesized and cloned into pET-28a (+), and the genes encoding US3 protein were synthesized and cloned into pGEX-6p-1 by GENEWIZ (Suzhou, China). The recombinant proteins were expressed in Escherichia coli BL21 and purified using BeaverBeads GSH and His tag according to the instructions. GST resins were incubated with purified GST-US3 protein at 4°C for 3 h and then with His-ANXA2 protein at 4°C overnight. The samples were washed with TBS more than five times, and proteins were eluted and subjected to WB with the indicated antibodies.

### Cell viability detection.

PK-15 or 3D4/21 cells were seeded onto 96-well plates, treated with indicated inhibitors at 37°C for 72 h, or transfected with siRNAs at 37°C for 36 h. The CCK-8 solution was added to each well, and the plates were incubated at 37°C for 1.5 h. The optical density at 450 nm (OD_450_) was measured using a microplate reader (BioTek, Winooski, VT, USA).

### Generation of polyclonal antibody targeting US3 of PRV.

The antigen US3 of PRV was expressed in E. coli BL21 and purified using BeaverBeads GSH. A polyclonal antibody was developed according to the methods previously studied (56), but by mice: 50 μg US3 was emulsified in Freund’s complete adjuvant and then injected into three ~5-week-old female BALB/c mice. After that, the immunization was repeated three times every 2 weeks after the initial vaccination with the emulsion mixture of US3 and incomplete Freund’s adjuvant. Two mice with the highest antibody titers against US3 protein were intraperitoneally boosted with 100 μg US3. After 3 days, one of them was sacrificed, and the supernatant of the whole blood was collected by centrifugation and stored at –80°C as PRV US3 polyclonal antibody.

### Evaluation of A2ti-1 and PP2 for their prophylactic and therapeutic efficacy in mice.

The method to test the prophylactic and therapeutic efficacy of A2ti-1 and PP2 in mice was taken from a previous study (55). For prophylactic efficacy, ~5-week-old BALB/c mice (*n* = 8) were administered intraperitoneally with either DMSO or A2ti-1 or PP2 at doses of 8 mg/kg at 96, 48, and 0 h before the challenge. For therapeutic efficacy, the mice (*n* = 8) were intraperitoneally injected with DMSO or A2ti-1 or PP2 at single doses of 8 mg/kg at 0, 24, and 72 h postchallenge. The survival rate was monitored daily for 7 days. At 4 days postchallenge, three mice from the therapeutic and control group were euthanized. Half the lungs and brains were fixed in universal tissue fixative (Servicebio, G1101) and stained by hematoxylin and eosin staining by Servicebio (Wuhan, China), the other half were collected and homogenized in PBS, and the viral DNAs were extracted by FastPure cell/tissue DNA isolation minikit before being determined by qPCR.

### Inhibitor treatments.

PK-15 or 3D4/21 cells were treated with noncytotoxic specific inhibitors or DMSO for 12, 24, and 36 h along with PRV at 37°C before subsequent experiments.

### PRV titration assay.

PK-15 cells were seeded in 96-well plates and overgrew the entire well. Virus samples were serially diluted from 10^−1^ to 10^−10^ in DMEM, used to inoculate cells at 37°C for 1 h, and then washed with PBS more than three times after removing the virus-DMEM mixture. The infected cells were cultured in 2% FBS in DMEM at 37°C with 5% CO_2_ for 24 to 48 h. The cytopathic effect (CPE) on PK-15 cells was counted under the microscope before calculating the 50% tissue culture infective dose (TCID_50_) by the Reed-Muench method.

### Immunofluorescence assay (IFA) and confocal microscopy.

For IFA and permeabilized staining, PK-15 or 3D4/21 cells were washed with ice-cold PBS more than three times, fixed with 4% PFA for 20 min, and permeabilized with 0.1% Triton X-100 in PBS for 10 min at RT. After being rinsed more than three times with ice-cold PBS, the cells were blocked with 3% bovine serum albumin (BSA) for 1.5 h at RT and incubated with the appropriate primary antibodies at 4°C overnight. Afterward, the cells were incubated with appropriate secondary antibodies for approximately 1.5 h, and the cell nuclei were stained with DAPI for about 10 min.

For surface staining, PK-15 cells were washed with ice-cold PBS and stained with primary antibodies at 4°C for about 2 h, fixed with 2% PFA for 20 min, and stained with appropriate secondary antibodies for approximately 1.5 h, followed by staining the cell nuclei with DAPI for about 10 min at RT. The fluorescent images were acquired by a confocal laser scanning microscope (Zeiss LSM700) with the confocal laser scanning setup (20×, 40×, or 63×) and were represented as a single slice of a stack from three independent experiments (57). The colocalization analyses were performed using the JaCoP plugin in ImageJ software according to previous research guidelines (28, 58, 59). Pearson’s correlation coefficient (>0.5) describes the correlation of the intensity distribution between channels. In addition, ImageJ software was also used to measure and analyze the single-channel fluorescence intensity (60, 61).

### Cell surface elution.

Since ANXA2 contains a calcium-regulated phospholipid binding domain, it can attach to negatively charged cell membrane phospholipids by calcium (62). Therefore, extracellular ANXA2 can be dissociated from the cell surface membrane by the extracellular calcium-chelating agent EGTA (31). PK-15 cells were seeded in 10-cm plates for 24 h and infected with different multiplicities of infection (MOIs) of PRV or transfected with different concentrations of US3- and US3^mt^-p3×Flag expression plasmid. After being infected or transfected for a predetermined time, the cells were washed more than three times with ice-cold PBS. Then cells were eluted with PBS buffer containing 20 mM EGTA (31) at 4°C for 30 min. The eluates were collected, centrifuged at 1,200 rpm for 10 min, and detected by WB.

### Attachment, internalization, and release assay.

For the PRV attachment assay, the siNC or siRNA-transfected 3D4/21 and PK-15 (wild-type and knockout type) cells were infected with PRV and incubated at 4°C for 1 h. After adsorption, the unbound viruses were rinsed with ice-cold PBS more than five times, and the relative level of cell-bound viral DNA was quantified by qPCR (63). For the PRV internalization assay, the cells were infected with PRV and incubated at 4°C for 1 h. After adsorption, the unbound viruses were rinsed with ice-cold PBS more than five times, and the inoculated cells were transferred to 37°C for 1 h to allow viral internalization. The viruses not entering were removed with sodium citrate (Beyotime Biotechnology, ST368), followed by more than five washes with ice-cold PBS, and the entering viral DNA was analyzed by qPCR for UL54. For the PRV release assay, PK-15 (wild-type and knockout type) cells were infected with PRV and incubated at 37°C for 1 h. The unbound viruses were washed away, and the inoculated cells were transferred to 37°C. The cells and supernatants were harvested at 6, 12, and 24 hpi. The intracellular and extracellular virus titers were evaluated, and the ratio of TCID_50_ represented the release efficiency.

### Ethics statement.

All experiments were performed according to the Chinese Regulations on Laboratory Animals – The Guidelines for the Care of Laboratory Animals (Ministry of Science and Technology of the People’s Republic of China). All animal experiments were approved by the Animal Experiment Committee of Henan Academy of Agricultural Sciences with approval No. LLSC410118. This study did not involve endangered or protected species.

### Statistical analysis.

All of the data are presented as group means ± standard error of the mean (SEM). Statistical analyses and calculations were performed using GraphPad Prism software with the unpaired two-tailed Student’s *t* test. Statistical significance in the figures is indicated by asterisks: *, *P < *0.05; **, *P < *0.01; ***, *P < *0.001; ns, not significant, *P* > 0.05.
