# Supplementary material for: Pseudorabies Virus Regulates the Extracellular Translocation of Annexin A2 To Promote Its Proliferation
Source: J Virol. 2023 Feb 14;97(3):e01545-22. doi: 10.1128/jvi.01545-22 (PMC10062141; doi:10.1128/jvi.01545-22)
Supplement: Supplemental file 2 — Table S2. Download jvi.01545-22-s0002.pdf, PDF file, 0.08 MB [file jvi.01545-22-s0002.pdf]

Table S2: Primers used in this study.

Primer Name	Sequence (5'-3')	Purpose
siRNA-NC-F	UUCUCCGAACGUGUCACGUTT	Knockdown of ANXA2 gene in porcine cells, negative control
siRNA-NC-R	ACGUGACACGUUCGGAGAATT	
siANXA2-212#-F	GCCUUUGCCUACCAAAGAATT	Knockdown of ANXA2 gene in porcine cells
siANXA2-212#-R	UUCUUUGGUAGGCAAAGGCTT	
siANXA2-546#-F	GCUCUGUCAUUGAUUAUGATT	Knockdown of ANXA2 gene in porcine cells
siANXA2-546#-R	UCAUAAUCAAUGACAGAGCTT	
siANXA2-670#-F	CCUCCAGAAAGUAUUUGAATT	Knockdown of ANXA2 gene in porcine cells
siANXA2-670#-R	UUCAAAUACUUUCUGGAGGTT	
pig GAPDH-F	CCTTCCGTGTCCCTACTGCCAAC	qPCR primers for pig GAPDH
pig GAPDH-R	GACGCCTGCTTCACCACCTTCT	
pig ANXA2-F	CCTGCTCAGTATGACGCT TCT	qPCR primers for pig ANXA2
pig ANXA2-R	TCTGGAGCAGATGATCTCAAT	
PRV UL54-F	TGCAGCTACACCCTCGTCC	qPCR primers for PRV UL54
PRV UL54-R	TCAAAACAGGTGGTTGCAGTAAA	
pig sgRNA1-F	CACCGCGCTGAAGTCAGCCTTGTC	Encoding sgRNA for Cas9 targeting exon 5 of ANXA2 to generate ANXA2-KO PK15 cells
pig sgRNA1-R	AAACGACAAGGCTGACTTCAGCGC	
pig sgRNA2-F	CACCGTCAGCCTTGTCCGGCCACC	Encoding sgRNA for Cas9 targeting exon 5 of ANXA2 to generate ANXA2-KO PK15 cells
pig sgRNA2-R	AAACGGTGGCCGGACAAGGCTGAC	
PK15 ANXA2-F	GTTGTGTTATTTGTAACCTGCACT	Amplifying and identifying wild-type and ANXA2-KO PK15 cells
PK15 ANXA2-R	AATCTTTTTTAAGGAGGAAGTGTAATC	
PRV sgRNA24-F	CACCGCGACGAGATCCTGTACTCGG	Encoding sgRNA for Cas9 targeting US3 gene to generate PRV ΔUS3 virus
PRV sgRNA24-R	AAACCCGAGTACAGGATCTCGTCGC	
PRV sgRNA72-F	CACCGAGATCATCATCGACGGCGA	Encoding sgRNA for Cas9 targeting US3 gene to generate PRV ΔUS3 virus
PRV sgRNA72-R	AAACTCGCCGTCGATGATGATCTC	
PRV sgRNA654-F	CACCGCTACGACACCAAGGTCGACG	Encoding sgRNA for Cas9 targeting US3 gene to generate PRV ΔUS3 virus
PRV sgRNA654-R	AAACCGTCGACCTTGGTGTCGTAGC	
PRV sgRNA659-F	CACCGCGCGACCGCTACGACACCA	Encoding sgRNA for Cas9 targeting US3 gene to generate PRV ΔUS3 virus
PRV sgRNA659-R	AAACTGGTGTCGTAGCGGTCGCGC	
PRV US3-F	CAAGATCTATGGCCGACGCCGGAATCCC	Amplifying and identifying wild-type and PRV ΔUS3 virus
PRV US3-R	CGGAATTCTTTATACGGTCCACATTCC	
